# Screening cardiovascular risk factors of diabetes patients in the primary diabetes clinics

**DOI:** 10.1097/MD.0000000000026722

**Published:** 2021-07-30

**Authors:** Lingwang An, Yanlei Wang, Chenxiang Cao, Tao Chen, Yonghong Zhang, Linhui Chen, Shuhong Ren, Manni Tang, Fenglian Ma, Xianglan Li, Shuang Yuan, Wenhui Zhao, Yaujiunn Lee, Jianzhong Xiao

**Affiliations:** a Beijing Ruijing Diabetes Hospital, Beijing; b Beijing Tsinghua Changgung Hospital, School of Clinical Medicine, Tsinghua University; c Chengdu Ryan Diabetes Hospital, Chengdu; d Taiyuan Diabetes Hospital, Taiyuan; e Lanzhou Ruijing Diabetes Hospital, Lanzhou; f Heilongjiang Ruijing Diabetes Hospital, Harbin, Heilongjiang, China; g Lee's Clinic, No. 130, Min-Zu Rd, Pingtung, Taiwan.

**Keywords:** coronary disease, glycemic control, risk factors, type 2 diabetes mellitus

## Abstract

To evaluate the atherosclerotic cardiovascular diseases (ASCVD) risk factors in type 2 diabetes patients from the primary diabetes clinics for further comprehensive intervention in China.

A cross-sectional study was conducted in 5 primary diabetes chain hospitals in Beijing, Lanzhou, Harbin, Chengdu, and Taiyuan in continuous patients with type 2 diabetes from March 2016 to December 2019. The data collected at the first visit were analyzed, and proportions of patients reached the targets (glycosylated hemoglobin [HbA_1_c] < 7%, blood pressure < 130/80 mm Hg, and low-density lipoprotein cholesterol [LDL-C] < 2.6mmol/l) were calculated. The clinical characteristics and the associated factors with achievement in HbA_1_c, blood pressure, and LDL-C targets were analyzed.

A total of 20,412 participants, including 11,353 men (55.6%), with an average age of (59.4 ± 10.4) years were enrolled. Nearly 95% diabetes had one or more ASCVD risk factors other than hyperglycemia. The control rates of HbA_1_c, blood pressure, and LDL-C were 26.5%, 27.8%, and 42.6%, respectively. Only 4.1% patients achieved all 3 targets. Nearly 95% patients had one or more ASCVD risk factors other than hyperglyciemia. Diabetes duration, family history, and overweight/obesity were associated with the number of aggregated ASCVD risk factors. The patients with older age, no overweight/obesity, not smoking, less ASCVD risk factors, and having special diabetes care insurance (Chengdu) were associated with a higher control rates.

To deal with poor control status, global management of ASCVD risk factors, weight loss, and smoking cessation must be emphasized in the primary diabetes care settings. Special diabetes care insurance should be advocated.

Current ClinicalTrial.gov protocol ID NCT03707379. Date of Registration: October 16, 2018. https://clinicaltrials.gov.

**Key Points**

- The prevalence of ASCVD risk factors was high and the control rates were low in the primary diabetes care hospitals in China.
- Overweight/obesity, smoking, and poor diabetes care insurance were associated with the aggregated ASCVD risk factors and lower control rate.

## 1 Introduction

Diabetes mellitus become an epidemic disease around world including China in the last 3 decades.^[1,2]^ In the latest national survey,^[3]^ the estimated prevalence of diabetes was 10.9% among adult Chinese. Atherosclerotic cardiovascular diseases (ASCVD) are the major cause of death for diabetes patients.^[4–6]^ Meanwhile, ASCVD risk factors such as obesity, hypertension, dyslipidemia, and others are very common in patients with diabetes. The art-of-state studies demonstrate that intensive control of hyperglycemia, hypertension, and hypercholesterolemia markedly reduces the events of ASCVD in patients with diabetes.^[7–10]^ Steno-2 study^[11]^ indicates comprehensive ASCVD risk control is the most effective approach for complication prevention in type 2 diabetes (T2DM), which is the cornerstone for diabetes management. Ten years ago, the China Cardiometabolic Registries 3B (CCMR-3B) study,^[12]^ covering 104 hospitals in 6 geographical regions, including 25,817 diabetes patients, illustrates that the control rates of blood pressure, blood lipid, and blood glucose were 28.4%, 42.9%, and 47.8%, respectively. Only 1 in 18 patients reached all these 3 targets for blood pressure, low-density lipoprotein cholesterol (LDL-C), and glycosylated hemoglobin (HbA_1_c). In contrast, the improvement of global control of ASCVD risk factors is witnessed in the developed countries such as United States, which lead to a remarkable reduction of diabetes complication, especially ASCVD.^[13]^

Confronted with the huge number of diabetes and other non-communicable chronic diseases patients in China, primary care institutions are encouraged to be “primary” by the government. Diabetes is an important chronic disease. As we know, chronic diseases care models have been developed and implemented elsewhere.^[14–19]^ Regarding to the diabetes care in primary care setting, a shared care model was developed in Taiwan, and it has been proved to be effective.^[20]^ This model emphasized a continuous care provided by doctors, diabetes educators, and dietitians as a team to improve global control for the ASCVD risk factors. The model was introduced from Taiwan by the Ruijing Diabetes Chain Hospitals, including 5 diabetes specific primary care hospitals in 5 cities in the mainland China where different diabetes care insurance models exist. As the baseline investigation, this cross-sectional study was to evaluate the ACSVD risk factors among T2DM patients who visit primary diabetes clinics at the first time. In addition, the control status and the associated factors were analyzed.

## 2 Materials and methods

### 2.1 Study design

This was a cross-sectional, observational, multicenter study based on routine clinical practice. Participants were enrolled at diabetes hospitals from 5 big cities in China.

### 2.2 Estimated sample size

In CCMR-3B study,^[21]^ the comprehensive compliance rate of blood lipid, blood pressure, and blood glucose in patients completed secondary education and below with diabetes was 7.5%, and that in patients completed college and above with diabetes was 9%. In order to have a 90% probability of showing a statistically significant difference (using *P* < .05) in proportions, the total number of people in our study was at least 842. The data were continuous registration case records from hospitals. The sample size was much bigger than statistics requirement.

### 2.3 Study population

Patients attending Ruijing diabetes hospitals (a chain primary, private, and disease-specific hospital system) were the candidates. Five hospitals from Beijing, Lanzhou, Harbin, Chengdu, and Taiyuan were included. The data collected continuously from March 2016 to December 2019. Patients aged between 18 and 80 years old with diagnosis of T2DM based on the WHO diagnostic criteria in 1999^[22]^ were included in our study.

### 2.4 Exclusion criteria

Patients who had serious heart, liver, lung, kidney, and other organ dysfunction, being pregnant, or had been diagnosed as other types of diabetes were excluded.

### 2.5 Ethics

This study was approved by the ethics committee of Tsinghua Changgung Hospital (No. [2016] 004).

### 2.6 Data collection

Demographic data, education level, smoking status, individual medical history (hypertension, dyslipidemia, and cardiovascular disease), family history of diabetes mellitus, and treatments (oral antidiabetic agents, insulin, antihypertensive, lipid lowering, and antiplatelet agents) of participants were collected through face-to-face interview. The patient's height, body weight, and waist circumference were measured. Blood pressure was measured 3 times with a 3-minute interval by electronic sphygmomanometer after sitting at least 5 minutes. The mean value of the blood pressures was recorded. Blood samples were collected after an overnight, 10 to 14 hours fasting, and the laboratory tests were conducted in the local hospital, including liver function, renal function, fasting blood glucose, HbA_1_c, and lipid profiles. HbA_1_c was measured by high-performance liquid chromatography using the Automatic Glycohemoglobin Analyzer ADAMS A_1_c HA-8180 (Arkray, Japan) or MQ-2000 PT HbA_l_c analyzer (Huizhong, Shanghai, China), which had achieved the second level reference method certification of glycosylated hemoglobin of International Clinical Chemistry Committee. Blood lipid, liver, and kidney function were measured by automated analysis (Beckman counter AU5800). All the labs had participated local province lab quality control as required by the authority. All data were automatically downloaded from the hospital information system.

### 2.7 Diseases definition

Hypertension was defined as blood pressure ≥ 140/90 mm Hg, or taking antihypertensive drugs or self-reported previous diagnosis by health care professionals. Hyperlipidemia was defined as LDL-C ≥ 2.6 mmol/l, taking lipid-lowering drugs or self-reported previous diagnosis by health care professionals. Overweight was defined as body mass index (BMI) ≥ 24 kg/m^2^, and obesity was defined as BMI ≥ 28 kg/m^2^.^[23]^

The control target was <7% for HbA_1_c (A), <130/80 mm Hg for blood pressure (B), and <2.6 mmol/l for LDL-C (C).^[24]^

### 2.8 Statistical analysis

The general data were described for 5 individual hospitals, and the characters of patients were analyzed according to the aggregated numbers of ASCVD risk factors, namely hypertension, hyperlipidemia, overweight/obesity, and smoking. Kolmogorov–Smirnov test (K-S) was used to test the normality of data. The data of normal distribution was presented by mean and standard deviation, otherwise median and quartile were used. One-way analysis of variance and General Linear Model were used to compare the mean value of multiple groups. The Chi-Square test was used to compare the rates of multiple groups. Spearman's rank correlation was used to analyze the relationship between numbers of ASCVD risk factors, waist circumference, BMI, and control rates. Multivariate logistic regression analysis was used to analyze the associated factors with whether or not reaching all 3 HbA_1_c (A), blood pressure (B), and LDL-C (C) (ABC) targets. Variables with *P* < .2 in univariate analyses and gender, age, diabetes duration, smoking were also included in the multivariate phase for adjustment. *P* < .05 was defined as statistically significant. SPSS 26.0 (SPSS Inc., Chicago, IL) was used for statistical analysis.

## 3 Results

A total of 20,412 patients were investigated, including 11,353 men (55.6%) and 9059 women (44.4%), with an average age of (59.4 ± 10.4) years (Table 1). The control rates of HbA_1_c, blood pressure, and LDL-C were 26.5%, 27.8%, and 42.6%, respectively. Only 4.1% patients achieved all 3 ABC targets. Among 5 hospitals, 36.4% of patients in Chengdu achieved the HbA_1_c target, which was the highest, comparing with the lowest percentage in Lanzhou at 18.3%. Patients in Chengdu also had the highest blood pressure control rate (45.7%), while patients in Harbin had the lowest one (17.9%). The highest LDL-C control rate was seen in Lanzhou at 49.2%. In contrast, the lowest control rate was in Beijing (35.2%). The percentage for patients reached all 3 targets in Chengdu was the highest (9.3%), while it was the lowest in Harbin (1.9%) among 5 hospitals.

**Table 1:** General characteristic of participants in different clinics.

	Total	Beijing	Lanzhou	Harbin	Chengdu	Taiyuan	*P* value (overall)
Cases, n	20,412	3047	3500	7567	3251	3047	
Age (yrs), mean ± SD	59.4 ± 10.4	57.3 ± 10.8	61.0 ± 9.9	58.8 ± 10.5	61.0 ± 10.0	59.3 ± 10.2	<.001
Gender (male, n [%])	11,353 (55.6%)	1825 (59.9%)	2037 (58.2%)	4104 (54.2%)	1812 (55.7%)	1575 (51.7%)	<.001
Diabetes duration (yrs), m ± SD	8.8 ± 6.7	9.5 ± 7.4	9.1 ± 5.8	7.9 ± 6.8	9.4 ± 6.3	9.4 ± 6.6	<.001
Education							<.001
Below high school (n [%])	8236 (40.3%)	1021 (33.5%)	1684 (48.1%)	1992 (26.3%)	1956 (60.2%)	1583 (52.0%)	
High school and advance (n [%])	7796 (38.2%)	1274 (41.8%)	1587 (45.3%)	2640 (34.9%)	1138 (35.0%)	1157 (38.0%)	
Smoking							<.001
Current (n [%])	2339 (11.5%)	594 (19.5%)	552 (15.8%)	290 (3.8%)	593 (18.2%)	310 (10.2%)	
Past or never (n [%])	18,073 (88.5%)	2453 (80.5%)	2948 (84.2%)	7277 (96.2%)	2658 (81.8%)	2737 (89.8%)	
WC (male, cm)	91.0 ± 8.8	92.1 ± 9.1	89.8 ± 8.8	92.8 ± 8.7	87.6 ± 7.1	91.4 ± 9.0	<.001
WC (female, cm)	87.3 ± 9.3	87.0 ± 9.1	87.1 ± 9.6	88.5 ± 9.5	85.2 ± 7.6	87.5 ± 9.6	<.001
BMI (kg/m^2^)	25.2 ± 3.4	25.8 ± 3.6	24.4 ± 3.1	25.6 ± 3.4	24.5 ± 3.1	25.1 ± 3.4	<.001
HbA_1_c (%)	8.4 ± 2.0	8.1 ± 1.9	9.0 ± 2.3	8.4 ± 1.9	7.9 ± 1.9	8.7 ± 2.1	<.001
SBP (mm Hg)	132.1 ± 18.6	131.8 ± 16.8	129.3 ± 14.4	135.6 ± 20.0	127.9 ± 14.2	131.4 ± 23.1	<.001
DBP (mm Hg)	79.1 ± 10.5	80.2 ± 10.0	77.2 ± 8.9	82.3 ± 11.0	74.9 ± 8.5	76.6 ± 10.5	<.001
LDL-C (mmol/l)	2.76 (2.19–3.36)	2.95 (2.33–3.60)	2.61 (2.10–3.22)	2.83 (2.28–3.40)	2.66 (2.02–3.29)	2.66 (2.11–3.24)	<.001
HbA_1_c < 7% (n [%])	5417 (26.5%)	1015 (33.3%)	639 (18.3%)	1915 (25.3%)	1182 (36.4%)	666 (21.9%)	<.001
BP < 130/80 mm Hg (n [%])	5677 (27.8%)	777 (25.5%)	1052 (30.1%)	1356 (17.9%)	1486 (45.7%)	1006 (33.0%)	<.001
LDL-C < 2.6 mmol/l (n,(%))	8693 (42.6%)	1074 (35.2%)	1721 (49.2%)	2917 (38.5%)	1531 (47.1%)	1450 (47.6%)	<.001
Reaching 2 targets (n [%])	4348 (21.3%)	641 (21.0%)	734 (21.0%)	1270 (16.8%)	991 (30.5%)	712 (23.4%)	<.001
Reaching 3 targets (n [%])	836 (4.1%)	135 (4.4%)	131 (3.7%)	143 (1.9%)	301 (9.3%)	126 (4.1%)	<.001

It was found that patients with more ASCVD risk factors (hypertension, hyperlipidemia, overweight or obesity, and smoking) tended to have an older age, a longer duration of diabetes, a larger waist circumference and a higher BMI (Table 2). In addition, more people had diabetes family history as the risk factors aggregated. Obviously, they tend to have higher HbA_1_c, blood pressure, and LDL-C levels.

**Table 2:** Demographics, laboratory results, and control rates of T2DM sample stratified by the number of ASCVD risk factors.

	T2DM only	T2DM with 1 risk factor	T2DM with 2 risk factors	T2DM with more than 3 risk factors	*P* value (overall)
Cases, n	1127	4383	7554	7348	
Age (yrs), mean ± SD	57.9 ± 11.2	58.6 ± 10.6	59.5 ± 10.5	60.0 ± 10.0	<.001
Age groups					
≤50 (n [%]) (n = 3447)	241 (7.0%)	845 (24.5%)	1303 (37.8%)	1058 (30.7%)	<.001
>50, ≤65 (n [%]) (n = 10,750)	591 (5.5%)	2315 (21.5%)	3890 (36.2%)	3954 (36.8%)	
>65 (n [%]) (n = 6215)	295 (4.7%)	1223 (19.7%)	2361 (38.0%)	2336 (37.6%)	
Gender (male (n [%]) (n = 11,353)	618 (54.8%)	2272 (51.8%)	4094 (54.2%)	4369 (59.5%)	<.001
Diabetes duration (yrs), m ± SD	7.9 ± 6.1	8.4 ± 6.4	8.7 ± 6.7	9.2 ± 6.9	<.001
Diabetes duration groups					<.001
≤5 (n [%]) (n = 6044)	364 (6.0%)	1322 (21.9%)	2272 (37.6%)	2086 (34.5%)	
>5, ≤10 (n [%]) (n = 4814)	271 (5.6%)	1045 (21.7%)	1794 (37.3%)	1704 (35.4%)	
>10 (n [%]) (n = 7382)	341 (4.6%)	1469 (19.9%)	2684 (36.4%)	2888 (39.1%)	
Education					.23
Below high school (n [%]) (n = 8236)	448 (5.4%)	1828 (22.2%)	3065 (37.2%)	2895 (35.2%)	
High school and above (n [%]) (n = 7796)	450 (5.8%)	1680 (21.5%)	2828 (36.3%)	2838 (36.4%)	
Smoking					<.001
Current (n [%]) (n = 2339)	0 (0.0%)	151 (6.5%)	614 (26.3%)	1574 (67.3%)	
Past or never (%) (n = 18,073)	1127 (6.2%)	4232 (23.4%)	6940 (38.4%)	5774 (31.9%)	
WC (male, cm)	84.3 ± 7.7	87.1 ± 7.8	90.7 ± 8.3	94.3 ± 8.3	<.001
WC (female, cm)	80.2 ± 6.4	83.1 ± 7.6	87.1 ± 8.7	91.8 ± 9.2	<.001
BMI (kg/m^2^)	21.7 ± 1.8	23.1 ± 2.8	25.0 ± 3.1	27.1 ± 2.9	<.001
Diabetes family history					<.001
Yes (n [%]) (n = 5687)	238 (4.2%)	1145 (20.1%)	2018 (35.5%)	2286 (40.2%)	
No (n [%]) (n = 13,920)	856 (6.1%)	3046 (21.9%)	5200 (37.4%)	4818 (34.6%)	
HbA_1_c (%)	8.4 ± 2.3	8.5 ± 2.2	8.4 ± 2.0	8.4 ± 1.8	.47
FBG (mmol/l)	9.6 ± 3.9	9.8 ± 3.9	9.8 ± 3.8	9.9 ± 3.7	.22
SBP (mm Hg)	119.5 ± 2.3	123.6 ± 2.2	131.8 ± 2.0	139.3 ± 1.8	<.001
DBP (mm Hg)	73.2 ± 3.9	74.9 ± 3.9	78.9 ± 3.8	82.7 ± 3.7	<.001
Total cholesterol (mmol/l)	4.17 (3.70–4.64)	4.74 (4.05–5.56)	5.00 (4.28–5.80)	5.20 (4.44–5.96)	<.001
LDL-C (mmol/l)	2.11 (1.77–2.35)	2.50 (2.04–3.14)	2.79 (2.22–3.37)	3.01 (2.51–3.55)	<.001
Triglyceride (mmol/l)	1.20 (0.90–1.70)	1.45 (1.05–2.10)	1.66 (1.20–2.40)	1.87 (1.34–2.67)	<.001
HbA_1_c < 7% (n [%])	349 (31.0%)	1283 (29.3%)	2001 (26.5%)	1784 (24.3%)	<.001
BP < 130/80 mm Hg (n [%])	591 (52.4%)	1966 (44.9%)	2067 (27.4%)	1053 (14.3%)	<.001
LDL-C < 2.6 mmol/l (n [%])	1127 (100.0%)	2455 (56.0%)	3109 (41.2%)	2002 (27.2%)	<.001
Reached 3 targets (n [%])	195 (17.3%)	329 (7.5%)	206 (2.7%)	106 (1.4%)	<.001

Among patients without other ASCVD risk factors, the control rates of HbA_1_c, blood pressure, LDL-C, and all ABC factors were 31.0%, 52.4%, 100%, and 17.3%, respectively. The control rates were lower in patients with more ASCVD risk factors aggregated (Table 2).

When the control rates stratified by treatment used, namely insulin injection, antihypertensive, and lipid-lowering medicine, the control rates of HbA_1_c, blood pressure (BP), and LDL-C in patients under treatment were much lower than these in patients who did not (Table 3). With the increase of age, the control of blood lipid was better and blood pressure was worse. The blood glucose control of the elderly patients was better than that of the young and middle-aged patients (Table 4). Patients with older age, lower BMI, non-smoking, no insulin injection, without hypertension or hyperlipidemia, lived in Chengdu (with special diabetes care insurance) had higher control rate of all ABC goals (Table 5).

**Table 3:** The patient number reached goal and control rates stratified by treatment (n [%]).

	Total	Non-insulin	Insulin	Non-antihypertensive	Antihypertensive	Non-lipid lowering	Lipid lowering
HbA_1_c < 7%	5417 (26.5%)	3896 (33.1%)	1521 (17.6%)^∗∗^	3958 (25.9%)	1459 (28.4%)^∗∗^	4080 (26.6%)	1337 (26.3%)
BP < 130/80 mm Hg	5677 (27.8%)	3350 (28.5%)	2327 (26.9%)^∗^	4606 (30.1%)	1071 (20.9%)^∗∗^	4337 (28.3%)	1340 (26.3%)^∗∗^
LDL-C < 2.6 mmol/L	8693 (42.6%)	5008 (42.5%)	3685 (42.7%)	6350 (41.6%)	2343 (45.7%)^∗∗^	6707 (43.8%)	1986 (39.0%)^∗∗^
Reached 3 targets	836 (4.1%)	627 (5.3%)	209 (2.4%)^∗∗^	660 (4.3%)	176 (3.4%)^∗∗^	656 (4.3%)	180 (3.5%)^∗^

**Table 4:** The patient number reached goal and control rates stratified by age, gender, and diabetes duration (n [%]).

	BP < 130/80 mm Hg	HbA_1_c < 7%	LDL-C < 2.6 mmol/l	Reaching 3 goals
Age (yrs)				
≤50	1133 (32.9%)	1449 (42.0%)	732 (21.2%)	124 (3.6%)
>50, ≤65	3031 (28.2%)	4453 (41.4%)	2884 (26.8%)	442 (4.1%)
>65	1513 (24.3%)	2791 (44.9%)	1801 (29.0%)	270 (4.3%)
*P* value	<.001	<.001	<.001	.21
Sex				
Male	3031 (26.7%)	5222 (46.0%)	2968 (26.1%)	453 (4.0%)
Female	2646 (29.2%)	3471 (38.3%)	2449 (27.0%)	383 (4.2%)
*P* value	<.001	.15	<.001	.40
Diabetes duration (yrs)				
<1	586 (30.0%)	726 (37.1%)	555 (28.4%)	67 (3.4%)
≥1, <5	1158 (28.4%)	1686 (41.3%)	1336 (32.7%)	184 (4.5%)
≥5, <10	1420 (29.5%)	2099 (43.6%)	1289 (26.8%)	221 (4.6%)
≥10	1970 (26.7%)	3245 (43.9%)	1675 (22.7%)	299 (4.0%)
*P* value	.001	<.001	<.001	.11

**Table 5:** Associated factors of patients reaching all 3 ABC targets.

Potential predictor	Univariate regression		Multivariate regression		Multivariate regression adjusted^∗^	
	OR (95% CI)	*P* value	OR (95% CI)	*P* value	OR (95% CI)	*P* value
Age (every 10 yrs increase)	1.07 (1.00–1.14)	.06	1.12 (1.04–1.21)	.003	1.11 (1.02–1.21)	.01
Gender (male vs female)	0.94 (0.82–1.08)	.40			0.92 (0.77–1.09)	.32
Diabetes duration (every 2 yrs increase)	0.98 (0.90–1.07)	.71			1.04 (0.93–1.15)	.51
Education (high school or above vs below high school)	0.91 (0.78–1.06)	.22	1.13 (0.96–1.32)	.15	1.18 (0.99–1.40)	.06
BMI (≥24 kg/m^2^ vs *b* < 24 kg/m^2^)	0.60 (0.52–0.69)	<.001	0.75 (0.64–0.88)	<.001	0.77 (0.65–0.91)	.002
Current smoking vs non-smoking and withdrawal	0.99 (0.80–1.23)	.93			0.72 (0.56–0.94)	.02
Clinic in other cities vs clinic in Harbin						
Beijing	1.10 (0.91–1.33)	.31	3.26 (2.42–4.41)	<.001	3.74 (2.72–5.14)	<.001
Lanzhou	0.89 (0.74–1.08)	.25	1.35 (1.02–1.78)	.04	1.64 (1.21–2.22)	.001
Chengdu	3.17 (2.74–3.67)	<.001	5.43 (4.24–6.95)	<.001	6.18 (4.73–8.08)	<.001
Taiyuan	1.01 (0.83–1.23)	.91	2.60 (1.96–3.46)	<.001	2.82 (2.07–3.83)	<.001
Insulin therapy vs OAD or TLC	0.44 (0.38–0.52)	<.001	0.42 (0.35–0.51)	<.001	0.41 (0.34–0.50)	<.001
History of hypertension (yes vs no)	0.53 (0.46–0.61)	<.001	0.65 (0.54–0.77)	<.001	0.64 (0.53–0.76)	<.001
History of hyperlipidemia (yes vs no)	0.18 (0.15–0.20)	<.001	0.17 (0.14–0.20)	<.001	0.17 (0.14–0.20)	<.001

## 4 Discussion

Our study provided a first overview of the prevalence and the control status of ASCVD risk factors in these diabetes specific primary care settings in China.

Nearly 95% diabetes patients had one or more ASCVD risk factors (hypertension, dyslipidemia, overweight or obesity, and smoking) other than hyperglycemia, and 73% of them had 2 or more. These were similar to the results reported in literatures. Woodard et al^[25]^ found that 92.2% diabetes patients had one or more comorbidities (hypertension, ischemic heart disease, hyperlipidemia). REACTION study^[26]^ found that 88.8% diabetes patients had at least 1 additional condition (hypertension, hyperlipidemia, hypothyroidism, hyperthyroidism, or renal insufficiency), and 53.2% of patients had 2 or more comorbidities. Wang et al^[27]^ reported that 1 or more chronic conditions (a total of 52 other chronic diseases including hypertension, hyperlipidemia, and coronary heart disease) experienced by 71% diabetes patients in communities.

In consistent with the REACTION research,^[26]^ our study showed that the number of ASCVD risk factors increased with age, diabetic duration, waist circumference, and BMI. In addition, the risk factors numbers increased in patients with diabetic family history just as reported in previous literatures.^[28–31]^

In this study, the control rate of blood pressure, blood lipid, and blood glucose of diabetes patients was lower. Only 26.5% patients achieved HbA_1_c target. The control rate of blood glucose was lower as the number of risk factors increased. The control rate was similar to that (25.9%) reported in Shaanxi Province in western China.^[32]^ However, it was much lower than the national-wide data (36.7%^[26]^ to 47.8%^[2,12,33]^). The discrepancy might be due to those data either from a tertiary/secondary hospital^[12]^ with better health care resources or from epidemiological study^[33]^ that included a higher proportion newly diagnosed diabetes patients. The achievement of HbA_1_c control was much lower than that from Americans (55.5%) reported in 2009 to 2010 NHANES survey^[34]^ and from Spain in 2009 (56.1%).^[35]^ In our study, only 27.8% and 42.6% of diabetes patients achieved blood pressure < 130/80 mm Hg and LDL-C < 2.6mmol/l, respectively. These were similar to those reported in CCMR-3B study (28.4% and 42.9%)^[12]^ and in Spain (31.7% and 37.9%).^[35]^ However, these were also much lower than those reported in the United States (52.8% and 54.4%).^[34]^ One explanation was that people with blood pressure between 130/80 mm Hg and 140/90 mm Hg were not treated as hypertension. This speculation was supported by only 52.4% patients with type 2 diabetes only reached BP goal. Regarding the global control rate, only 4.1% of patients reached all 3 ABC targets in our study. It was as low as that reported from Shaanxi Province (4.5%)^[32]^ and it was even lower than that in CCMR-3B study (5.6%)^[12]^ 10 years ago. The proportion of patients reached all 3 ABC targets was only about one-third of the proportion in Spain (12.1%),^[35]^ or was one-sixth of the proportion (24.9%) in the United States^[34]^ and Canada (21%).^[36]^ The unsatisfactory control status may also be due to the selection bias, that is, patients with poor controlled blood glucose might prefer to visit the specialized diabetes clinics instead of general hospital. Details of comparison with previous studies were shown in Table 6. It was interesting to find the difference among these 5 hospitals. Particularly, patients in Chengdu had the best control of HbA_1_c, blood pressure, and all 3 ABC targets. Better health insurance policy may contribute to the achievement in Chengdu, where the patients diagnosed with diabetes were granted a special quota for diabetes care by local municipal insurance agency.

**Table 6:** Results of individual or combined treatment goals achieved for the T2DM patients in different studies and also stratified according to sex and age.

Studies	BP < 130/80 mm Hg (%)	HbA_1_c < 7% (%)	LDL-C < 2.6 mmol/l (%)	BP < 130/80 mm Hg, HbA_1_c < 7%, and LDL-C < 2.6 mmol/l (%)	BP < 140/90 mm Hg (%)	BP < 140/90 mm Hg, HbA_1_c < 7%, and LDL-C < 2.6 mmol/l (%)	Study design
Xu^[2]^		39.7					A national wide, complex, multistage, probability sampling design
Gao^[26]^		36.7					National wide, community-based study
Ji^[12]^	28.4	47.7	42.9	5.6			Patients from endocrinology, cardiology, nephrology, and internal medicine clinics in Tier 1–3 hospitals
Lv^[33]^							Newly diagnosed T2DM patients from Tier 1–3 hospitals
<65		33.5	37		44.3	11.1	
≥65		47.8	39.4		44.9	13.5	
Xu^[32]^		25.9		4.5			6 tertiary hospitals across Shaanxi province
Vinagre^[35]^	31.7	56.1	37.9	12.1			All patients with T2DM treated at the Catalan Health Institute, who were nearly free of charge
<65	33.3	51.8	32.8	11.9			
≥65	30.9	58.5	40.6	12.1			
Male	32	55.8	41.3	13.3			
Female	31.4	56.5	34.2	9.9			
Braga^[36]^	54	53	64	21			Primary care physicians were instructed to enroll T2DM patients
Wong ND^[34]^	34.2–52.8	35–55.5	37–54.4	2.5–24.9			NHANES 1999–2010
Our study	27.8	26.5	42.6	4.1	60.9	8.5	
<65	29.3	25.5	41.6	4	63.4	8.4	
≥65	24.3	29	44.9	4.3	55.3	8.9	
Male	26.7	26.1	46	4			
Female	29.2	27	38.3	4.2			

We found that patients with older age, shorter duration of diabetes, lower BMI, non-smoking, and oral hypoglycemic agent, had a higher proportion of achieving all 3 therapeutic goals. These were consistent with the findings from other studies.^[12,32]^ Among them, the relationship between age and combined target rate was more complex (Tables 7 and 8). Although older patients had more comorbidities and their blood pressure was more difficult to control, they had better compliance, lower BMI, lower smoking rate, lower diabetes family history rate, and better management of blood lipids and blood glucose (Table 7). Our data suggested that the failure of pancreatic function (insulin therapy), overweight or obesity, and un-healthy life-style (smoking) were key to impact a global control of ASCVD risk factors. Thus, lifestyle intervention such as stopping smoking and losing weight played an important role in the control of ASCVD risk factors.

**Table 7:** General characteristic and control rates of participants stratified by different age groups.

Age (yrs)	18–53.5 (n = 5103)	53.6–60.4 (n = 5103)	60.5–66.4 (n = 5105)	66.4–80 (n = 5101)	*P* value (overall)
Gender (male, n [%])	3384 (66.3%)	2811 (55.1%)	2717 (53.2%)	2441 (47.9%)	<.001
Diabetes duration (yrs), m ± SD	5.9 ± 4.9	8.2 ± 6.1	9.6 ± 6.6	11.5 ± 7.5	<.001
Education					<.001
Below high school (n [%])	1499 (18.2%)	1833 (22.3%)	2320 (28.2%)	2584 (31.4%)	
High school and above (n [%])	2430 (31.2%)	2108 (27.0%)	1706 (21.9%)	1552 (19.9%)	
Smoking					<.001
Current (n [%])	838 (35.8%)	626 (26.8%)	537 (23.0%)	338 (14.5%)	
Past or never (%)	4265 (23.6%)	4477 (24.8%)	4568 (25.3%)	4763 (26.4%)	
WC (cm)	89.3 ± 9.6	89.2 ± 8.9	89.4 ± 9.1	89.4 ± 9.1	.76
BMI (kg/m^2^)	25.5 ± 3.7	25.2 ± 3.3	25.1 ± 3.3	25.0 ± 3.2	<.001
Diabetes family history					<.001
Yes (n [%])	1708 (30.0%)	1546 (27.2%)	1416 (24.9%)	1017 (17.9%)	
No (n [%])	3237 (23.3%)	3344 (24.0%)	3470 (24.9%)	3869 (27.8%)	
HbA_1_c < 7% (n [%])	1145 (22.4%)	1308 (25.6%)	1509 (29.6%)	1455 (28.5%)	<.001
BP < 130/80 mm Hg (n [%])	1618 (31.7%)	1491 (29.2%)	1324 (25.9%)	1244 (24.4%)	<.001
LDL-C < 2.6 mmol/l (n [%])	2134 (41.8%)	2097 (41.1%)	2147 (42.1%)	2315 (45.4%)	<.001
Reaching 3 targets (n [%])	193 (3.8%)	207 (4.1%)	213 (4.2%)	223 (4.4%)	.50

**Table 8:** General characteristics of participants in different clinics stratified by numbers of ABC targets reached.

	Total	Could not reach any target	Reaching 1 target	Reaching 2 targets	Reaching 3 targets	*P* value (overall)
Cases (n [%])	20,412 (100.0%)	6645 (32.6%)	8583 (42.0%)	4348 (21.3%)	836 (4.1%)	
Age (yrs), mean ± SD	59.4 ± 10.4	59.2 ± 10.5	59.5 ± 10.4	59.4 ± 10.3	60.1 ± 10.0	.066
Gender (male, n [%])	11,353 (55.6%)	3523 (53.0%)	4892 (57.0%)	2485 (57.2%)	453 (54.2%)	<.001
Diabetes duration (yrs), m ± SD	8.8 ± 6.7	9.0 ± 6.7	8.9 ± 6.8	8.3 ± 6.5	8.5 ± 6.4	<.001
Education						.33
Below high school (n [%])	8236 (40.3%)	2524 (38.0%)	3502 (40.8%)	1827 (42.0%)	383 (45.8%)	
High school and advance (n [%])	7796 (38.2%)	2470 (37.2%)	3256 (37.9%)	1739 (40.0%)	331 (39.6%)	
Current smoking (female, n [%])	146 (1.6%)	50 (1.6%)	55 (1.5%)	38 (2.0%)	3 (0.8%)	.24
Past smoking (female, n [%])	24 (0.3%)	4 (0.1%)	12 (0.3%)	6 (0.3%)	2 (0.5%)	
Current smoking (male, n [%])	2193 (19.3%)	667 (18.9%)	935 (19.1%)	499 (20.1%)	92 (20.3%)	.87
Past smoking (male, n [%])	365 (3.2%)	108 (3.1%)	149 (3.0%)	92 (3.7%)	16 (3.5%)	
WC (male, cm)	91.0 ± 8.8	92.2 ± 8.6	91.3 ± 8.8	89.4 ± 8.7	87.7 ± 8.0	<.001
WC (female, cm)	87.3 ± 9.3	88.7 ± 9.3	87.4 ± 9.3	85.7 ± 8.9	83.7 ± 7.5	<.001
BMI (male, kg/m^2^)	25.3 ± 3.2	25.7 ± 3.3	25.3 ± 3.2	24.8 ± 3.1	24.2 ± 2.9	<.001
BMI (female, kg/m^2^)	25.1 ± 3.6	25.6 ± 3.6	25.1 ± 3.6	24.6 ± 3.5	24.0 ± 3.2	.017
HbA1c (%)	8.4 ± 2.0	9.2 ± 1.8	8.5 ± 2.0	7.5 ± 2.0	6.2 ± 0.5	<.001
SBP (mm Hg)	132.1 ± 18.6	139.2 ± 20.2	132.1 ± 16.9	124.1 ± 15.3	116.0 ± 8.6	<.001
DBP (mm Hg)	79.1 ± 10.5	83.5 ± 9.6	79.1 ± 10.3	74.2 ± 9.3	69.1 ± 5.7	<.001
LDL-C (mmol/l)	2.76 (2.19–3.36)	3.29 (2.94–3.81)	2.58 (2.10–3.21)	2.21 (1.83–2.53)	2.01 (1.63–2.33)	<.001
HbA_1_c < 7% (n [%])	5417 (26.5%)	0 (0.0%)	1974 (23.0%)	2607 (60.0%)	836 (100.0%)	<.001
BP < 130/80 mm Hg (n [%])	5677 (27.8%)	0 (0.0%)	2250 (26.2%)	2591 (59.6%)	836 (100.0%)	<.001
LDL-C < 2.6 mmol/l (n [%])	8693 (42.6%)	0 (0.0%)	4359 (50.8%)	3498 (80.5%)	836 (100.0%)	<.001

In Table 3, the patients who used antihypertensive, hypoglycemic, and lipid-lowering drugs had lower control rates of blood pressure, blood glucose, and blood lipid, which should be explained that the patients with more comorbidities and higher ABC index but the control rate was low. Therefore, for patients with more comorbidities, management should be strengthened and ASCVD risk factors should be strictly controlled. Moreover, no hypertensive drugs used in those patients with blood pressure between 130/80 and 140/90 mm Hg may be also one reason for low control rate for BP. That cholesterol lowering medicine prescription did not increase the rate attaining all 3 targets in this study might be due to the tendency of Chinese patients to take lower doses of statins. In other literatures, non-Hispanic Whites rather than Black/African Americans, and Filipino and Hispanics/Latinos,^[37]^ men rather than women^[38]^ were more likely to achieve all 3 goals. This might also be due to the different doses of statins used in those patients.

This study has been the first large-scale study from the primary care setting ever in China. The limitation was the selection bias. Because patients choosing primary diabetes clinics in our study, were less educated compared with the CCMR-3B study, with more ASCVD risk factors compared with REACTION research (Table 9). This indicates that our patients’ compliance might be poor, and ABC index control was even worse. Patients with severe complications and those have well-controlled risk factors may not be proportionally recruited in our study. In addition, this study lacked individual information of the medical insurance status and economic situation, which would also affect the control rate of ASCVD risk factors. The degree of education is associated with the socioeconomic status, and we found that people attended college and above had a better control rate (Table 9).

**Table 9:** Numbers of ASCVD risk factors and individual or combined treatment goals achieved for the T2DM patients in different studies and also stratified according to educational level.

Studies	Total	T2DM only (%)	T2DM with 1 risk factor (%)	T2DM with 2 risk factors (%)	T2DM with 3 risk factors and more (%)	BP < 140/80 mm Hg (%)	HbA_1_c < 7% (%)	Total serum cholesterol <4.5 mmol/l (%)	Reached 3 targets (%)
Gao N^[26]^							36.7		
Less than secondary	3256 (63.5%)	10.2	35.4	43.9	10.6				
Secondary	1323 (25.8%)	13.4	35.5	39.6	11.5				
Postsecondary	547 (10.7%)	11.9	37.1	40.2	10.8				
Tao X (CCMR-3B)^[21]^									
Illiteracy	1695 (6.7%)								∼7.1
Primary education	5667 (22.3%)								∼7.5
Secondary education	11,936 (46.9%)								∼7.5
College and above	6156 (24.2%)								9
Our study		5.6	21.9	36.8	35.7	39.2	27.1	34.2	5.1
Less than secondary	1999 (12.5%)	5.7	23.3	39	32.1	45.1	23.1	37.2	5.7
Secondary	12,325 (76.9%)	5.6	21.9	36.6	35.9	38.3	27	33.3	4.8
Postsecondary	1708 (10.7%)	5.5	20.4	35.1	39	38.8	32.8	36.8	6

## 5 Conclusion

ASCVD risk factors were common and not well controlled in patients with type 2 diabetes. Longer duration of diabetes, smoking, and overweight/obesity were associated with more ASCVD risk factors aggregated. The more comorbidities aggregated in patients were associated with a worse global control. Special medical insurance policy may contribute to the better control achievement. In order to prevent ASCVD, global management of risk factors, education focus on smoke cessation, and weight loss should be emphasized. An affordable insurance policy was also critical.

## Author contributions

**Data curation:** Chenxiang Cao.

**Formal analysis:** Yanlei Wang.

**Funding acquisition:** Jianzhong Xiao.

**Investigation:** Lingwang An, Tao Chen, Yonghong Zhang, Linhui Chen, Shuhong Ren, Fenglian Ma, Xianglan Li, Shuang Yuan.

**Methodology:** Jianzhong Xiao.

**Project administration:** Yaujiunn Lee, Jianzhong Xiao.

**Supervision:** Manni Tang, Wenhui Zhao.

**Writing – original draft:** Yanlei Wang.

**Writing – review & editing:** Jianzhong Xiao.
